# Transformative Impact of Artificial Intelligence on Internal Medicine: Current Applications, Challenges, and Future Horizons for Urban Health

**DOI:** 10.14789/ejmj.JMJ25-0019-R

**Published:** 2025-11-18

**Authors:** WATARU FUJITA, AKIRA SAKAMOTO, EIICHIRO SATO, TOMOHIRO KANEKO, NOBUYUKI KAGIYAMA

**Affiliations:** 1 Department of Cardiovascular Biology and Medicine, Juntendo University Graduate School of Medicine, Tokyo, Japan; 1 Department of Cardiovascular Biology and Medicine, Juntendo University Graduate School of Medicine, Tokyo, Japan

**Keywords:** artificial intelligence, internal medicine, medical diagnosis, healthcare technology, urban health

## Abstract

Artificial intelligence (AI) is rapidly transforming internal medicine by enhancing diagnostic accuracy, enabling personalized treatment, and optimizing patient management. As of August 2024, the U.S. Food and Drug Administration has authorized nearly 950 AI/ML-enabled medical devices, while an American Medical Association survey reported that 66% of physicians already incorporate AI into clinical practice. This review provides a comprehensive overview of AI's expanding role across internal medicine, highlighting its applications in medical interviews, text-based communication, and the interpretation of core diagnostic modalities such as electrocardiography, chest X-ray, and auscultation. While several FDA-approved AI tools are already integrated into clinical workflows, many technologies remain at the research or proof-of-concept stage, with validation often limited to retrospective or controlled trial settings.

The transformative potential of AI is particularly relevant in urban healthcare, where population density, limited resources, and disproportionate burdens of chronic and lifestyle-related diseases underscore the need for innovative solutions. AI can mitigate physician shortages, streamline care in overburdened systems, and support equitable access to diagnostics and treatment in metropolitan areas. Key technologies, including machine learning, deep learning, and large language models, are critically examined, along with emerging innovations such as EHR-based foundation models.

Despite its promise, AI integration raises ethical, legal, social, and regulatory challenges, including algorithmic bias, data privacy, validation standards, and workforce adaptation. This paper explores these multifaceted aspects, emphasizing the importance of collaborative efforts to ensure responsible and equitable implementation, ultimately aiming to improve patient outcomes and public health in the digital era.

## Introduction

Internal medicine serves as the foundation of adult healthcare, encompassing the diagnosis, treatment, and long-term management of a wide spectrum of acute and chronic diseases. In recent years, the field has faced growing challenges due to increasingly complex patient presentations, the rise in multimorbidity, and rapid demographic shifts—particularly the accelerated aging of populations in urban centers such as Tokyo. These trends have intensified the need for more efficient, integrated, and patient-centered care models that emphasize prevention and early intervention. Amid these pressures, artificial intelligence (AI) has emerged as a transformative technology capable of addressing systemic inefficiencies and clinical complexity. Core AI domains—such as machine learning (ML), deep learning (DL), natural language processing (NLP), and large language models (LLMs)—have demonstrated strong capabilities in analyzing vast clinical datasets, supporting diagnostic and therapeutic decisions, and facilitating personalized care. In this review, we explicitly distinguish between technologies that are FDA-cleared and implemented in clinical practice, and those that remain investigational, thereby clarifying their current stage of readiness for practical adoption. In particular, AI is showing great promise in enhancing fundamental diagnostic modalities, including electrocardiography (ECG), chest X-ray (CXR), and even auscultation. These technologies offer the potential to improve diagnostic accuracy, reduce clinician burden, and optimize resource allocation in busy clinical settings. In countries like Japan, the convergence of a super-aged society, rising healthcare costs, and urban healthcare workforce shortages has accelerated the adoption of AI and other advanced technologies. In densely populated metropolitan areas, these challenges are magnified by high patient volumes, uneven distribution of healthcare resources, and socioeconomic disparities that contribute to unequal access to care. As such, AI is increasingly seen not only as a tool for innovation but also as a practical response to these pressing urban public health challenges, where it can enhance diagnostic efficiency, optimize resource allocation, and improve equitable healthcare delivery. This review aims to provide a comprehensive overview of the current and potential roles of AI across the spectrum of internal medicine. Special emphasis is placed on urban healthcare contexts, where AI is particularly well- positioned to mitigate the growing burden of chronic disease and improve care delivery. By critically evaluating existing applications, identifying limitations, and exploring future directions, this review seeks to contribute to the strategic and responsible integration of AI into internal medicine.

## AI-enhanced diagnostic capabilities in internal medicine

### AI in medical interviews and clinical reasoning

The patient interview is a cornerstone of clinical diagnosis, and AI is beginning to play a transformative role in this critical interaction. The evolution from structured, questionnaire-based systems to sophisticated LLMs marks a significant advancement in AI-assisted medical interviewing. Early systems, such as Ubie, utilized question flowcharts based on Bayesian models to assist in medical history taking^1)^. More recently, advanced LLMs like ChatGPT and the Articulate Medical Intelligence Explorer (AMIE) have demonstrated the capacity to conduct initial interviews, analyze symptoms, and present differential diagnoses^2)^. Preliminary evaluations indicate modest, setting-dependent reductions in consultation time in controlled or semi-structured environments, while real-world effects remain variable; therefore, prospective validation is warranted. For instance, the latest version of GPT-4 was reported to accurately diagnose a significant proportion of cases based on medical history alone, comparable to physician performance in some contexts^3)^. AMIE, an LLM-based AI system optimized for diagnostic dialogue, has shown superior diagnostic accuracy and empathetic communication compared to primary care physicians in OSCE-format validations, though these were with simulated patients^4)^.

Beyond history taking, LLMs are exhibiting emerging capabilities in clinical reasoning. A systematic review and network meta-analysis of LLMs in answering clinical research questions indicated that models like ChatGPT-4o perform strongly on objective questions, while human experts still tend to excel in arriving at the top one or three diagnoses^5)^. Other models like Claude 3 Opus have shown strength in broader diagnostic considerations, and Gemini in triage and classification^5)^. This suggests a potential collaborative role, where LLMs assist in broadening diagnostic possibilities or handling specific types of clinical queries.

For example, in a patient presenting with non- specific fatigue and mild shortness of breath, an LLM might highlight not only common conditions such as anemia or viral infection but also suggest the possibility of rarer entities such as amyloidosis or early pulmonary hypertension. This kind of expanded differential, generated early in the diagnostic process, can help clinicians remain vigilant for less obvious but clinically significant conditions.

### Applications of LLMs in diagnosis

Large language models (LLMs) have shown strong potential in supporting diagnostic reasoning by synthesizing complex patient histories, summarizing EHRs, and integrating multimodal clinical data^5)^. They can assist clinicians in generating differential diagnoses, identifying rare conditions, and providing evidence-based treatment suggestions. Their versatility across both structured and unstructured data sources positions LLMs as a powerful adjunct in modern diagnostic workflows. Ongoing studies continue to evaluate their real-world accuracy, efficiency, and integration into clinical decision support systems.

### AI in the interpretation of foundational diagnostic modalities

AI is significantly enhancing the information gleaned from established diagnostic tests widely used in internal medicine. A common theme emerging across these modalities is AI's capacity to identify subtle patterns or derive quantitative measures that often extend beyond routine human perception or are challenging to standardize consistently. This positions AI not merely as an assistant for existing interpretive tasks but as an enabler of new diagnostic insights from familiar tests, effectively acting as a "super-sensor" for the internist.

#### Electrocardiography (ECG) beyond cardiology

The ECG, a ubiquitous and non-invasive tool, is being reimagined through AI, extending its diagnostic utility far beyond traditional cardiac assessments. AI-ECG models are demonstrating the ability to detect signatures of various systemic and non- cardiac conditions that are relevant to internal medicine practice.

AI algorithms have demonstrated the ability to detect latent paroxysmal atrial fibrillation from sinus rhythm ECGs with high accuracy. For instance, a study validated an AI model that identified underlying paroxysmal AF from 12-lead ECGs in sinus rhythm, achieving an area under the curve (AUC) of 0.87^6)^. Similarly, AI has been employed to estimate left ventricular ejection fraction from ECG data, a parameter traditionally assessed via echocardiography. In a large-scale study, an AI-enabled ECG model predicted increased left ventricular filling pressure with an AUC of 0.911, comparable to echocardiographic assessments^7)^.

AI models have shown good diagnostic accuracy in identifying hyperkalemia (pooled sensitivity 0.856, pooled specificity 0.788) and hypokalemia (pooled sensitivity 0.824, pooled specificity 0.724) from ECG recordings^8)^. This capability is particularly valuable in critical care settings for frequent monitoring and for home monitoring of patients with end-stage renal disease. However, it is important to note that many studies in this area suffer from a high risk of bias in patient selection, potentially overestimating diagnostic accuracy^8)^.

AI-ECG is making inroads in the detection of metabolic disorders. For instance, AI models can predict hyperthyroidism with Area Under the Curve (AUC) values exceeding 0.86 and also offer prognostic information regarding mortality and heart failure risk in these patients^9)^.

A notable development is the AIRE-DM tool, which analyzes routine ECGs to predict the risk of developing Type 2 Diabetes Mellitus (T2DM) up to ten years in advance with approximately 70% accuracy, a figure that improves when combined with genetic and clinical data^10)^. Importantly, such diagnostic advances directly inform subsequent AI- driven management strategies. For example, individuals identified as high risk by AI-ECG can then be enrolled into continuous glucose monitoring (CGM)-integrated platforms, where AI algorithms provide real-time personalized insulin dosing advice, lifestyle recommendations, and long-term monitoring. This seamless transition from AI-enabled early risk detection to AI-driven chronic disease management exemplifies the transformative potential of AI to reshape the entire care continuum.

**Figure 1:**
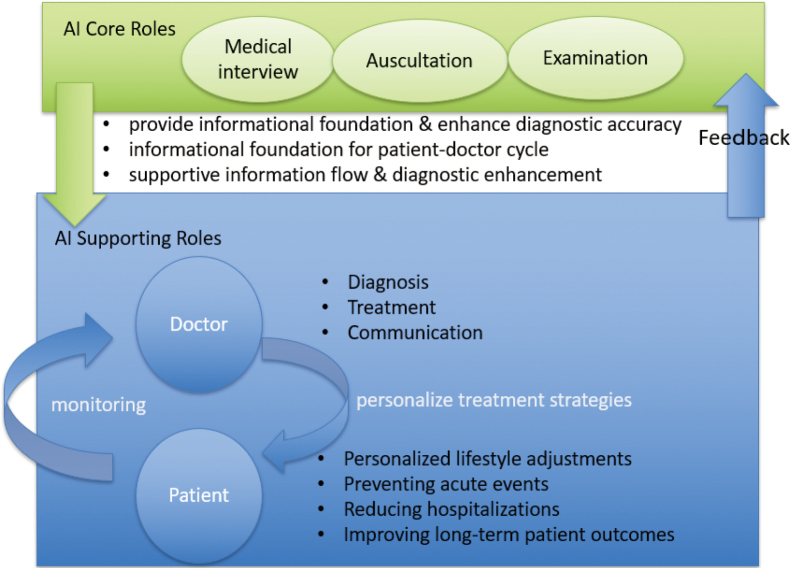
Core applications form an informational foundation that continuously supports subsequent AI roles This figure illustrates the main application domains of artificial intelligence (AI) in internal medicine. Specifically, it includes medical interview support, diagnostic modalities (electrocardiography, chest X-ray, and auscultation), remote monitoring, chronic disease management, and ethical considerations. The arrows represent the flow of AI-assisted data interpretation and clinical decision support, visually emphasizing the integration of AI into both diagnostic and therapeutic pathways. The arrow from AI Core Roles to AI Supporting Roles was revised to emphasize that core applications, such as AI-assisted interviews, not only enhance diagnostic accuracy but also provide an informational foundation for the entire patient-doctor cycle, thereby sustaining a continuous and supportive relationship between core and supporting roles.

#### Chest X-ray

The chest X-ray remains a fundamental diagnostic tool in internal medicine. AI is unlocking new levels of information from this classic test by detecting subtle changes that may be missed by the human eye.

AI algorithms excel in identifying a wide array of abnormalities on CXRs, including pneumonia, interstitial lung disease (ILD), lung cancer (nodules/ masses), pneumothorax, consolidation, and pleural effusion. The performance of AI in CXR analysis has been shown to match or even surpass that of experienced radiologists in certain tasks.

A significant advancement is the development of AI models like CXR-Lung-Risk, which can predicted respiratory disease mortality from a CXR with a hazard ratio of 2.01. Before clinical application, however, these results are promising, it is important to note that most evidence to date comes from retrospective analyses using curated datasets, which may not fully capture real-world diversity. The prognostic accuracy of such models can be further enhanced through longitudinal analysis of serial CXRs; however, robust external validation, prospective trials, and deployment in varied clinical environments remain essential to confirm generalizability. This perspective moves AI beyond simple abnormality detection towards providing valuable prognostic insights, while acknowledging the current limitations that must be addressed before widespread adoption. The clinical potential of AI in CXR is underscored by the growing number of FDA- cleared AI devices for computer-assisted triage (CAT) and computer-aided detection (CAD)^11)^. As of February 2025, a substantial portion of FDA- authorized AI medical devices were in radiology, with many targeting chest imaging^9)^.

#### AI in auscultation: Advancements in analyzing lung and bowel sounds

Auscultation, a traditional diagnostic skill, is being revitalized by AI, which offers methods to quantify auditory findings and improve diagnostic accuracy.

AI algorithms can detect and classify adventitious lung sounds such as wheezes, rhonchi, and crackles with high sensitivity. The field of "audiomics," leveraging AI to analyze respiratory sounds, is emerging as a source of non-invasive biomarkers for various pulmonary conditions including COVID-19, tuberculosis, ILD, asthma, and COPD. Meta-analyses of machine learning models for pediatric lung sound analysis, often using CNNs, have reported high pooled sensitivity and specificity for wheeze detection (0.902 and 0.955, respectively) and abnormal lung sound detection (0.907 and 0.877, respectively)^12)^. However, these studies are often limited by dataset heterogeneity and potential biases^12)^.

AI is also being applied to the analysis of bowel sounds, which can be indicative of gastrointestinal disorders. AI combined with wearable sensors, such as smart T-shirts embedded with microphones, enables automated bowel sound event spotting^13)^. Deep learning models like EffUNet have achieved an AUC of ≥ 0.83 in classifying Inflammatory Bowel Disease (IBD) versus healthy controls based on bowel sound characteristics^13)^. Pre-trained models such as HuBERT and Wav2Vec 2.0 have demonstrated superior performance in detecting and classifying various bowel sound patterns compared to traditional methods^14)^. This is particularly important as manual auscultation of bowel sounds is often subjective and limited by short observation times.

## AI-driven therapeutic and management strategies

### Enhancing patient monitoring

The proliferation of wearable sensors, Internet of Things (IoT) devices, and smartphones has created an unprecedented opportunity for continuous, real-time patient monitoring outside traditional healthcare settings^15)^. AI algorithms are essential for transforming the raw data streams from these devices into clinically actionable insights. AI can extract meaningful patterns from complex movement data (e.g., from inertial measurement units - IMUs), physiological signals (e.g., heart rate, ECG, oxygen saturation), and environmental factors to aid in diagnostics, monitor disease progression, and assess treatment efficacy^15)^.

AI combined with IMUs or video analysis is used to assess gait patterns for conditions like Parkinson's disease (e.g., VisionMD software^16)^), to evaluate frailty in heart failure patients , or to monitor general mobility and fall risk in the elderly.

Smartwatches equipped with ECG capabilities, when coupled with AI algorithms, can detect arrhythmias like atrial fibrillation, identify signs of left ventricular systolic dysfunction or myocardial infarction, and even predict heart failure rehospitalization.

AI-powered virtual assistants and mobile apps can help patients manage medications, track symptoms, adhere to lifestyle recommendations, and schedule appointments, thereby improving self- management of chronic conditions.

The benefits of AI-driven remote monitoring are particularly significant for home care, the management of chronic diseases, and providing healthcare in remote or underserved areas. Internet of Medical Things (IoMT) platforms, by enabling seamless data flow and analysis, have the potential to facilitate early disease detection and minimize delays in intervention^17)^. However, realizing this potential requires addressing substantial challenges, including data privacy and security, ensuring interoperability between diverse devices and systems (though standards like ISO/IEEE 11073 and HL7 FHIR are helping^17)^), and guaranteeing equitable access to these technologies, especially for medically marginalized communities who may face a digital divide or be disproportionately affected by biases in wearable sensor technology (e.g., inaccuracies in pulse oximeters for individuals with darker skin tones^15)^).

### AI in chronic disease management

AI is poised to revolutionize the management of chronic diseases by enabling a more holistic, proactive, and personalized approach, moving beyond episodic care to continuous health management.1 AI algorithms can analyze comprehensive patient health data, including EHRs, imaging, lab results, genetic information, and data from wearables, to develop personalized management plans and predict disease trajectories^18)^. Mobile health technologies and machine learning are currently key focal points in this domain^19)^. AI’s impact extends beyond accurate diagnosis to the orchestration of continuous, personalized management. For instance, an AI- ECG that predicts elevated diabetes risk enables downstream interventions such as integration with CGM data for real-time glucose monitoring, AI- assisted insulin dosing, and proactive lifestyle modification guidance. Similarly, AI-ECG tools that forecast heart failure rehospitalization risks seamlessly connect to smartwatch-based monitoring and teleintervention platforms, ensuring timely treatment adjustments. This linkage illustrates how diagnostic and management applications are not isolated but rather interconnected components of a transformative, end-to-end AI-enabled care pathway. Regarding COPD and asthma, predictive modeling for exacerbations, personalized treatment plans delivered via telemedicine platforms, and AI-assisted analysis of respiratory sounds for monitoring^20)^. Early detection of chronic kidney disease, through analysis of subtle data patterns, risk stratification for progression and complications, and AI-guided treatment recommendations^21)^. In patients with heart failure, AI-ECG for early risk prediction and smartwatch- based remote monitoring to prevent rehospitalizations. Importantly, a multicenter randomized controlled trial^22)^ demonstrated that mobile health-based teleintervention significantly improved clinical outcomes and reduced hospital readmissions, further reinforcing the clinical utility of these approaches.

The overarching impact of these AI-driven strategies, particularly the integration of AI with continuous data from wearables, IoT devices, and EHRs, is a fundamental shift in how internal medicine approaches patient care. Traditional chronic disease management often involves periodic clinical evaluations and reactive interventions when symptoms worsen. In contrast, AI enables a paradigm where continuous, real-time physiological and behavioral data are analyzed to detect subtle, early warning signs of disease onset or deterioration, often before these are apparent to the patient or clinician. For example, the AIRE-DM tool aims to predict diabetes risk years before clinical manifestation^10)^, and AI- smartwatch ECG systems endeavor to predict heart failure rehospitalizations^23)^. Importantly, these diagnostic insights represent the entry point into an integrated patient care pathway, directly informing personalized treatment and management strategies described in Chapter 3. This capability allows for preemptive interventions, personalized lifestyle adjustments, or timely modifications to therapy, potentially preventing acute events, reducing hospitalizations, and improving long-term patient outcomes.

## Barriers and future perspectives

### Ethical, legal, and social implications (ELSI)

The deployment of AI in internal medicine raises profound ELSI concerns that must be proactively addressed to ensure responsible innovation.

AI systems can inadvertently perpetuate or even amplify existing societal biases present in historical health data, leading to disparities in healthcare delivery and outcomes for different demographic groups^15)^. Bias can manifest at various stages: pre- processing (e.g., selection bias, measurement bias, representation bias), in-processing (algorithmic bias), and post-processing (evaluation bias)^24)^. A critical issue is "fairness drift," where an algorithm deemed fair at deployment may become biased over time due to changes in data distributions or patient populations, or as a result of model updating strategies^25)^. Continuous monitoring for fairness drift and adaptive mitigation strategies are therefore essential. Mitigation approaches include curating diverse and representative datasets, developing fairness-aware algorithms (e.g., re-sampling, re- weighting, adversarial debiasing, constraint-based optimization), implementing transparent model evaluation, and establishing clear lines of accountability^26)^.

The use of AI, particularly models trained on large-scale patient data from EHRs or wearables, raises significant data privacy and security concerns. Robust data governance frameworks, adherence to regulations like GDPR^27)^, HIPAA^28)^, and Japan’s APPI^29)^, and the use of privacy-enhancing technologies (e.g., federated learning*, differential privacy**, homomorphic encryption) are crucial. The deidentification of patient data is a key requirement, though achieving perfect deidentification that prevents re- identification, especially with rich datasets, remains a challenge.

Beyond data governance, AI applications in internal medicine must also comply with medical AI regulatory frameworks. These include the FDA’s software-as-a-medical-device (SaMD) framework, provisions under the EU AI Act addressing high-risk medical AI, and guidance from Japan’s Ministry of Health, Labour and Welfare (MHLW) on AI/ML-based medical devices^30-32)^. Collectively, these frameworks provide the foundation for ensuring transparency, safety, and accountability in the clinical adoption of AI.

The opacity of many advanced AI models is a major barrier to trust. XAI techniques are vital for providing transparency and enabling clinicians to understand, verify, and critically appraise AI-generated outputs^33)^. Public and patient trust is fundamental for the successful adoption of AI in healthcare. Studies indicate that while patients are open to AI as a supportive tool, they generally prefer human oversight in decision-making and value comprehensibility, data protection, and evidence of validation^34)^. Trust in AI is influenced by factors such as general attitudes towards technology, individual personality traits, and knowledge about AI^35)^.

### The evolving role of the internist

The integration of AI into internal medicine will inevitably reshape the role of the internist, requiring adaptation in skills and a transformation in medical education.

Physicians will need to develop a combination of digital competencies and enhanced critical human skills^36)^. Digital competencies include a basic understanding of AI principles, data analysis, the ability to critically evaluate AI-generated outputs, and the skill to detect errors or biases in AI-assisted medicine. Simultaneously, "soft" skills such as empathy, nuanced communication, complex clinical judgment (especially in situations of uncertainty or conflicting data), and ethical reflection become even more crucial as AI handles more data-intensive tasks.

The future is not one of AI replacing physicians, but rather AI augmenting their capabilities. Clinicians must become "conscious users" of AI, understanding its strengths and limitations, and integrating AI-derived insights into their broader clinical decision-making process while retaining ultimate responsibility

Medical school curricula and postgraduate training programs must adapt to prepare future internists for this AI-augmented environment. This includes incorporating education on AI fundamentals, data science, biostatistics, the ethics of AI, and methods for critically appraising AI tools and research^36)^. Initiatives like the AMA ChangeMedEd^®^ Artificial Intelligence in Health Care Series provide resources for this educational shift^36)^.

There are legitimate concerns about potential skill erosion ("deskilling") if physicians become over- reliant on AI, as well as the impact of AI on the patient-physician relationship. Ambiguity persists regarding the precise extent of AI understanding required by physicians, which may vary by specialty and role.

### Challenges specific to LLMs

Despite their promise, the use of LLMs in diagnosis and management is fraught with challenges. The "hallucination" problem, where LLMs generate plausible but incorrect or fabricated information, remains a significant concern^37, 38)^. Reliability and the need for vigilant clinician oversight are paramount^5)^. Compounding this, some research indicates that worse-performing LLMs can exhibit paradoxically higher confidence in their outputs, potentially misleading clinicians^39)^. The causes of medical hallucinations are multifaceted, stemming from issues in data quality, diversity, and the scope of training data, and require robust mitigation strategies including improved data curation, advanced training methods, and external knowledge integration like Retrieval-Augmented Generation (RAG)^37, 38)^. In addition to data-related factors, Transformer- based LLMs are fundamentally probabilistic next-token predictors without native symbolic reasoning or built-in truth verification, which can produce fluent yet logically inconsistent outputs—particularly under distribution shift or during long, compositional reasoning. Another concern is that advanced LLMs may exhibit cognitive biases similar to those observed in human clinicians, such as framing effects, primacy effects, and hindsight bias^40)^. The "personality" traits of LLMs, influenced by their training data and architecture, can further modulate these biases. This suggests that as LLMs become more sophisticated in their reasoning, their potential errors may shift from overt factual inaccuracies to more subtle, bias-driven misjudgments that are harder to detect.

Clinicians can actively mitigate some of these risks by employing specific prompting strategies, such as asking the LLM to challenge its own conclusions, provide evidence against a favored diagnosis, or generate alternative hypotheses^40)^. Awareness- based debiasing prompts, instructing the model to be mindful of cognitive biases, have also been evaluated, with effectiveness varying by LLM personality and architecture.

Ultimately, patient trust is a key factor. Current evidence suggests that while patients may value the information AI can provide, they tend to trust human expertise more for definitive diagnoses, particularly when explanations for diagnostic reasoning are provided^41)^.[Table t001]

**Table 1 t001:** Ethical, legal, and social issues (ELSI) in AI for internal medicine and proposed mitigation frameworks

ELSI concern	Specific manifestations in internal medicine	Potential harms/challenges	Mitigation strategies & role of XAI	Relevant snippets (Examples)
Algorithmic bias & fairness	Biased diagnostic algorithms (e.g., CXR, ECG, LLM-based diagnosis) underperforming for certain demographics; Inequitable risk stratification for chronic diseases (CKD, T2DM).	Exacerbation of health disparities; Misdiagnosis; Inappropriate treatment; Erosion of trust in minority groups.	Diverse & representative training datasets; Fairness-aware algorithms (pre-, in-, post-processing); Regular bias audits; Fairness drift monitoring; XAI-based bias detection (e.g., SHAP***, LIME****) with subgroup error auditing; reweighting/resampling and threshold optimization as needed. XAI for bias detection; Regulatory oversight.	
Data privacy & security	Breaches in EHR systems used for training foundation models; Unauthorized access to data from wearables/IoT devices; Misuse of sensitive patient information by LLMs.	Identity theft; Discrimination; Erosion of patient confidentiality; Legal liabilities.	Robust data governance; Privacy-enhancing techniques (federated learning*, homomorphic encryption); Strict adherence to GDPR/HIPAA; Secure deidentification methods; Patient consent protocols.	
Lack of transparency ("Black box")	Opaque decision-making in DL models for image analysis (endoscopy, radiology); Unclear reasoning in LLM diagnostic suggestions or prognostic models.	Difficulty in verifying AI outputs; Reduced clinician trust; Challenges in identifying error sources; Hindrance to accountability.	XAI techniques (SHAP***, LIME****, SHAP, GradCAM) to provide model interpretability; Model cards and datasheets; Transparent reporting of model development and validation.	33
Accountability & liability	Determining responsibility for medical errors when AI is involved (e.g., misdiagnosis by an AI-CDSS leading to patient harm).	Legal ambiguity; Difficulty in assigning blame (developer, institution, clinician); Potential for "responsibility gaps."	Clear regulatory guidelines on liability; Robust auditing trails for AI decisions (traceability); Professional guidelines for AI use; Insurance frameworks for AI-related incidents.	26
Digital divide & equity	Unequal access to AI-powered healthcare tools due to socioeconomic status, geographic location (urban vs. rural), or digital literacy; Biased wearables (e.g., pulse oximeters).	Widening health disparities; Reinforcement of existing inequities; Limited benefit of AI for marginalized communities.	Policies promoting equitable access; User-friendly design for diverse literacy levels; Community engagement in AI development; Public funding for AI in underserved areas.	5
Patient autonomy & trust	Patients feeling disempowered by AI-driven decisions; Lack of understanding or control over how their data is used by AI; Over-reliance on AI by clinicians impacting shared decision-making.	Reduced patient engagement; Erosion of patient-physician relationship; Non-adherence to AI-guided recommendations if trust is lacking.	Transparent communication about AI use; Patient education on AI capabilities and limitations; Mechanisms for patient input and consent; Ensuring human oversight in critical decisions.	34
Deskilling of professionals	Over-reliance on AI for diagnostic interpretation or treatment planning potentially leading to erosion of clinicians' core skills and critical thinking.	Reduced ability to function without AI; Challenges in managing complex/atypical cases not well-covered by AI; Potential for diagnostic errors if AI fails or is flawed.	Continuous medical education focusing on both AI literacy and core clinical skills; Emphasizing AI as an augmentation tool, not a replacement; Fostering critical appraisal of AI outputs.	

*Federated learning: Model training occurs locally at each institution; only model updates/parameters are shared and aggregated centrally, no raw patient data leave the site. **Differential privacy: A formal privacy technique that adds statistical noise to outputs so that information about any single individual cannot be inferred. ***SHAP: visualizes subgroup differences in feature contributions. ****LIME: provides local explanations to analyze causes of misclassification.

## Conclusion

AI is transforming internal medicine by enhancing diagnostics, enabling personalized treatment, and extending chronic disease management beyond hospital settings. Tools such as AI-assisted ECG, CXR, and auscultation improve accuracy and uncover patterns beyond human recognition. Predictive models and remote monitoring systems are helping shift care toward prevention and home-based management. However, challenges remain—algorithmic bias, lack of transparency, and inequities in digital access must be addressed through ethical oversight and inclusive design. To harness AI effectively, clinicians must balance its outputs with clinical judgment, and medical education must adapt to include AI literacy. Ultimately, successful implementation requires robust validation, ethical integration, and collaboration across disciplines to ensure AI supports—not replaces—human expertise in complex, real-world care. Within Japan, these developments must be considered in light of the universal health insurance system, which ensures equitable access but can slow the pace of innovation adoption, and the demanding physician work culture, where AI has the potential to reduce workload pressures. These unique factors underscore the need for implementation strategies tailored to Japan’s healthcare environment, thereby maximizing both safety and clinical utility.

## Author contributions

WF conceived the study, drafted the manuscript, and coordinated the overall process. AS contributed to the study design and provided critical feedback throughout the drafting process. NK supervised the entire project and gave final approval of the manuscript. ES and TK contributed to the interpretation of results and provided minor revisions. All authors read and approved the final manuscript.

## Conflicts of interest statement

NK was affiliated with a department endowed by grants Paramount Bed, received research grants from EchoNous. Inc. and AMI Inc., and received an honorarium from Novartis Japan, Otsuka Pharma, Eli Lilly, and Nippon Boehringer Ingelheim outside the submitted work.
